# Auditory and Visual Motion Processing and Integration in the Primate Cerebral Cortex

**DOI:** 10.3389/fncir.2018.00093

**Published:** 2018-10-26

**Authors:** Tristan A. Chaplin, Marcello G. P. Rosa, Leo L. Lui

**Affiliations:** ^1^Neuroscience Program, Biomedicine Discovery Institute and Department of Physiology, Monash University, Clayton, VIC, Australia; ^2^Australian Research Council (ARC) Centre of Excellence for Integrative Brain Function, Monash University Node, Clayton, VIC, Australia

**Keywords:** visual motion, auditory motion, audiovisual integration, primates, cerebral cortex

## Abstract

The ability of animals to detect motion is critical for survival, and errors or even delays in motion perception may prove costly. In the natural world, moving objects in the visual field often produce concurrent sounds. Thus, it can highly advantageous to detect motion elicited from sensory signals of either modality, and to integrate them to produce more reliable motion perception. A great deal of progress has been made in understanding how visual motion perception is governed by the activity of single neurons in the primate cerebral cortex, but far less progress has been made in understanding both auditory motion and audiovisual motion integration. Here we, review the key cortical regions for motion processing, focussing on translational motion. We compare the representations of space and motion in the visual and auditory systems, and examine how single neurons in these two sensory systems encode the direction of motion. We also discuss the way in which humans integrate of audio and visual motion cues, and the regions of the cortex that may mediate this process.

The natural world abounds with motion, making this a highly salient cue to guide animals in interacting with the environment. It is therefore not surprising that most, if not all brains have dedicated neural circuits for the perception of motion. In primates, the cerebral cortex contains a network of regions that are specialized for motion processing, but the systems for processing the motion of visual features and sounds are mediated by different brain regions, and underpinned by different physiological mechanisms. In this mini-review article, we will discuss the encoding of direction of motion in the visual and auditory systems, with emphasis on the cortical systems that are involved in translational motion, especially in azimuth (leftwards and rightwards motion), as this is the most common type of motion used in audiovisual integration studies.

## Encoding of Direction of Motion in the Activity of Cortical Neurons

**Figure 1:**
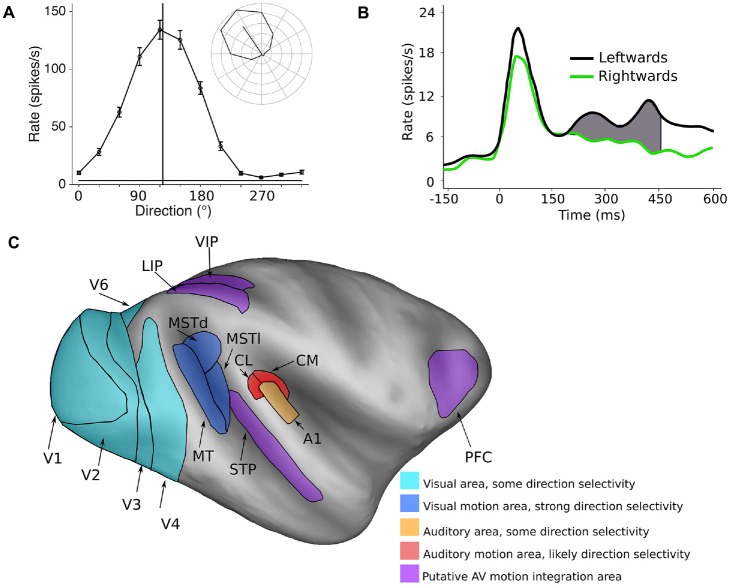
Encoding of direction of motion in the visual and auditory systems. **(A)** A typical visual direction tuning curve from a neuron in the marmoset visual cortex (area MT) in response to a moving dot stimulus (data from Chaplin et al., 2017). The vertical line indicates the preferred direction of motion, and the inset shows the mean spiking responses (with the spontaneous rate subtracted) in polar plot form, showing clear direction selectivity. **(B)** The temporal spiking response of a neuron in the macaque auditory cortex (A1) in response to a moving auditory stimulus. Here, the difference in firing rate between two directions of motion is quite modest, and is most obvious in the later part of the response. Redrawn with permission from the authors of Ahissar et al. (1992). **(C)** Inflated model of the macaque cerebral cortex showing some of the motion processing areas in the primate cerebral cortex (Van Essen, 2002; Van Essen and Dierker, 2007). Light blue areas: visual areas where a subpopulation of neurons shows direction selectivity, dark blue areas: visual motion processing areas MT, MSTd and MSTl, orange: A1, red: areas of the caudal auditory belt (CM, CL) which have been implicated in auditory motion processing, purple: areas that show auditory and visual motion responses and may be involved in integrating the two modalities.

Spatial features are represented in fundamentally different ways in the visual and auditory systems. In the visual system, most neurons have spatially defined receptive fields, which are ultimately defined by inputs from specific regions of the retina. Therefore, the responses of neurons in the visual system are inherently capable of coding the spatial location of visual stimuli, and in theory, could encode direction of motion by the sequential activation of populations of neurons with different receptive field locations. However, the visual system goes one step further, with direction of motion being explicitly represented at the level of the single cell. Specifically, the spiking (action potential) responses of neurons are tuned to the direction of moving stimuli, meaning that they are more active in response to a specific direction of motion compared to other directions (Dubner and Zeki, 1971; Baker et al., 1981; Maunsell and Van Essen, 1983a; Albright, 1984; Desimone and Ungerleider, 1986; Saito et al., 1986; Tanaka and Saito, 1989; Chaplin et al., 2017). Thus, direction selective neurons in the visual system can encode the direction of motion *within* their receptive fields. For example, Figure 1A shows the response of a direction tuned neuron: the neuron shows strong responses to motion towards the upper left quadrant, and progressively weaker responses for directions further away.

In contrast, most neurons in the auditory system respond to specific ranges of acoustic frequencies, since they ultimately receive inputs from defined regions of the cochlea. Thus, the auditory system needs to exploit other auditory cues to extract spatial information from the stimulus. The principal cues for locating sounds in the azimuth are binaural—interaural time differences (ITDs) and interaural level differences (ILDs; Middlebrooks and Green, 1991). Several brain regions are involved in the perception of sound location, and neurons in these regions can be tuned for ITDs or ILDs (Masterton et al., 1967; Rajan et al., 1990a,b; Semple and Kitzes, 1993a,b; Irvine et al., 1996; Tian et al., 2001; Woods et al., 2006; Miller and Recanzone, 2009; Grothe et al., 2010; Slee and Young, 2010; Kusmierek and Rauschecker, 2014; Keating and King, 2015; Lui et al., 2015; Mokri et al., 2015).

The encoding of the direction of auditory motion by the activity of single cortical neurons has not been studied extensively in primates—to our knowledge, there is only published study (Ahissar et al., 1992), in which they recorded spiking activity in the primary auditory cortex (A1) of monkeys. They found that while many cells (62%) in A1 code for the spatial location of stationary sounds, some cells (32%) also showed a preference for leftwards or rightwards direction of motion. However, the differences in responses were far less marked than those observed in direction selective cells in the visual system. There were only modest differences in firing rates, which were evident in the late part of the responses (Figure 1B). These results suggest that the encoding of the direction of motion of auditory stimuli is likely to be a much more distributed representation across a neuronal populations, compared to direction of motion encoding in the visual system (Cohen and Newsome, 2009), or that explicit encoding of auditory motion relies on other areas beyond A1.

## Visual Motion Processing Areas

The neural circuits for visual motion processing are among the best understood aspects of the structure and function of the primate cerebral cortex (Figure 1C, blue areas). The primary visual cortex (V1) is the first stage of visual processing in the cerebral cortex in which direction selectivity first appears, but only a small proportion of V1 neurons are direction selective (~15%, Yu et al., 2010; Yu and Rosa, 2014; Davies et al., 2016). Direction selective neurons have been observed in several other visual areas (Orban et al., 1986; Desimone and Schein, 1987; Felleman and Van Essen, 1987; Lui et al., 2005, 2006; Orban, 2008; Fattori et al., 2009; Li et al., 2013), but it is the middle temporal (MT) and medial superior temporal (MST) areas that appear to be most specialized for motion processing. The vast majority of cells in these regions are direction selective (MT ~85%: Allman and Kaas, 1971; Dubner and Zeki, 1971; Maunsell and Van Essen, 1983b; Albright, 1984; MST ~90%: Desimone and Ungerleider, 1986; Saito et al., 1986; Tanaka and Saito, 1989; Celebrini and Newsome, 1994; Elston and Rosa, 1997). Furthermore, it is known that damage to MT and MST results in motion perception impairments (Newsome and Paré, 1988; Pasternak and Merigan, 1994; Orban et al., 1995; Schenk and Zihl, 1997; Rudolph and Pasternak, 1999), and electrical stimulation of these regions can influence the perception of motion (Celebrini and Newsome, 1994, 1995; Salzman and Newsome, 1994; Britten and Van Wezel, 2002; Nichols and Newsome, 2002; Fetsch et al., 2014). Thus, a causal relationship has been established between neural activity in MT and MST and the perception of visual motion.

MST can be divided to two subregions: a lateral part (MSTl) involved in the perception of moving objects and smooth pursuit eye movements (Komatsu and Wurtz, 1988a,b; Eifuku and Wurtz, 1998), and dorsal part (MSTd), which is associated with the perception of complex motion patterns (Graziano et al., 1994; Mineault et al., 2012), especially self-motion (Saito et al., 1986; Komatsu and Wurtz, 1988a; Duffy and Wurtz, 1991; Duffy, 1998), and has a well described role in the integration of visual and vestibular motion cues (Gu et al., 2007, 2008). Differences between MT and MST have been well studied in monkeys, but in human studies these areas are typically grouped into a single region called the human MT complex (hMT+, Zeki et al., 1991; Huk et al., 2002), due to the spatial resolution limits of fMRI.

## Auditory Motion Processing Areas

In comparison to the visual system, the regions and circuitry of the cortex involved in auditory motion processing are not as well characterized (Figure 1C). While there is some evidence for motion sensitivity and direction selectivity in the A1 (Ahissar et al., 1992; Griffiths et al., 2000; Lewis et al., 2000), many human imaging studies have identified the planum temporale, a region of auditory cortex caudal to primary cortex, as being the key site for auditory motion processing (Baumgart et al., 1999; Pavani et al., 2002; Warren et al., 2002; Alink et al., 2012b). In agreement with these findings, a recent imaging study in macaques also found that the caudal regions of auditory cortex are differentially activated by auditory motion compared to stationary stimuli (Poirier et al., 2017). Furthermore, studies of humans with lesions to caudal auditory cortex have found deficits in auditory motion processing (Ducommun et al., 2004; Lewald et al., 2009; Thaler et al., 2016).

It remains controversial whether auditory motion perception relies on specialized motion detectors, similar to direction selective cells in the visual cortex (Perrott and Musicant, 1977), or utilizes “snapshots” of the current sound source location (Ahissar et al., 1992; Poirier et al., 2017), as several human imaging studies have reported there is no difference in cortical activation between stationary and moving stimuli (Smith et al., 2004, 2007; Krumbholz et al., 2005, 2007). Since neurons in the auditory system show sensitivity to localization cues (e.g., ITDs and ILDs), the perception of motion could be mediated by the sequential activation of neurons that code for adjacent spatial locations (Ahissar et al., 1992). In general, in the auditory system the integration of binaural cues for sound localization occurs at early subcortical stages of processing, such as the superior olivary complex, the nuclei of the lateral lemniscus and the inferior colliculus (Moore, 1991). In monkeys, the caudal part of auditory cortex encompasses the caudomedial (CM) and caudolateral (CL) areas of the auditory belt (Hackett et al., 1998; Kaas et al., 1999), and these are known to play a role in the localization of auditory stimuli (Recanzone et al., 2000; Tian et al., 2001; Woods et al., 2006; Miller and Recanzone, 2009; Kusmierek and Rauschecker, 2014). Therefore, the sensitivity of neurons in these areas to the location of static stimuli is a potential confound in auditory motion studies, as it can be difficult to distinguish true motion sensitivity from sensitivity to spatial location. For example, it has been suggested that apparent sensitivities to motion in the inferior colliculus could be explained by adaptation to stationary stimuli, which would result in reduced spiking activity for stationary stimuli compared to moving stimuli (Ahissar et al., 1992; Wilson and O’Neill, 1998; McAlpine et al., 2000; Ingham et al., 2001; Poirier et al., 2017). However, the recent imaging study by Poirier et al. (2017) did take steps to control for this effect in their choice of stimuli and regressions analyses, and still found that the caudal auditory cortex was differentially activated by auditory motion compared to static motion. Further electrophysiological studies in monkeys will be required to address the question of how auditory motion is encoded by the spiking activity of neurons in these regions.

The neural representation of auditory motion does not necessarily have to be located in purely auditory regions. Direct reciprocal connections between MT/MST and the auditory cortex have been identified in primates (Palmer and Rosa, 2006), and two recent electrophysiological studies (Chaplin et al., 2018; Kafaligonul et al., 2018) have reported evoked potentials in areas MT/MST in response to stationary auditory clicks. Two human imaging studies have reported that the hMT+ complex responds to auditory motion (Poirier et al., 2005; Strnad et al., 2013), but it has also been argued that observed auditory responses in hMT+ could be explained by localization errors (Jiang et al., 2014), and no study has found any evidence for spiking activity in response to auditory stimuli (moving or stationary) in the monkey MT complex. Furthermore, a case study of involving lesions of hMT+ did not find any impairment in the perception of auditory motion (Zihl et al., 1983). Thus, current evidence suggests that MT and MST are not involved in auditory motion processing.

## Integration of Auditory and Visual Motion Cues

Given the differences in the neural representation of motion in the auditory and visual systems, it is interesting to consider how the information from the two modalities could be combined to improve motion perception. Psychophysical studies have investigated audiovisual motion integration in humans using motion detection tasks, and have provided valuable insights into how auditory and visual motion can be integrated in the brain. Some of these studies have reported that humans perform better in audiovisual motion tasks compared to unimodal tasks, but there is disagreement as to whether this increase in performance is “statistically optimal” or the result of “probability summation.” When probability summation occurs, observers perform better on bimodal trials because they essentially have two chances to answer correctly—using either the visual or the auditory cue (Wuerger et al., 2003; Alais and Burr, 2004). When statistically optimal integration occurs, observers combine the information obtained by the different senses by weighting according to their reliability, to make optimal use of the information available (Meyer and Wuerger, 2001). Therefore, statistically optimal integration exceeds the performance of probability summation. Multisensory integration has shown be statistically optimal in other contexts (Ernst and Banks, 2002; Angelaki et al., 2009; Fetsch et al., 2009; Drugowitsch et al., 2014; Rohde et al., 2016).

It has been argued that statistically optimal integration of multisensory cues relies on neural computations occurring in early sensory cortex (e.g., MT/MST), rather than in higher-level areas (Ma et al., 2006; Beck et al., 2008; Bizley et al., 2016). In contrast, when multisensory integration is the result of probability summation, it may rely on higher-order areas (e.g., prefrontal or posterior parietal cortex, Alais and Burr, 2004; Bizley et al., 2016).

## Audiovisual Motion Integration in the Primate Cerebral Cortex

Human imaging studies and monkey electrophysiological/anatomical studies have suggested several candidate cortical regions for the integration of audiovisual motion. The human superior temporal sulcus is typically activated by moving audiovisual stimuli (Lewis et al., 2000; Baumann and Greenlee, 2007; von Saldern and Noppeney, 2013). This region likely corresponds to the superior temporal polysensory (STP) area of macaques (Bruce et al., 1981), and the presence of multisensory neurons in STP is well known (Bruce et al., 1981; Hikosaka et al., 1988; Watanabe and Iwai, 1991). STP is typically associated with processing more complex visual and auditory signals, such as faces and speech (Beauchamp, 2005) and biological motion (Oram and Perrett, 1994; Barraclough et al., 2005), especially in complex tasks (Meyer et al., 2011; Wuerger et al., 2012), but there is evidence of subregional specializations (Padberg et al., 2003).

The posterior parietal cortex may also be important for audiovisual motion integration, as areas in this complex have found to be active during audiovisual stimulation in humans (Baumann and Greenlee, 2007; Wuerger et al., 2012), and is thought play a key role in coordinating multisensory integration (Brang et al., 2013). Cells in the ventral intraparietal area (VIP) are known to respond to both visual motion (Cook and Maunsell, 2002; Kaminiarz et al., 2014) and auditory stimuli (Bremmer et al., 2001; Schlack et al., 2005). The lateral intraparietal area (LIP) has been demonstrated to be involved in the integration visual motion signals over time to form perceptual decisions (Roitman and Shadlen, 2002), and also responds to auditory stimulation (Grunewald et al., 1999; Linden et al., 1999). Therefore, it is possible that LIP could integration information from both senses, by preforming similar computations.

Integration could also occur at the level of the prefrontal cortex (PFC), as regions in the dorsolateral PFC (areas 8a, 45 and 46) are known to receive inputs from MT and MST (Lewis and Van Essen, 2000; Reser et al., 2013) as well as caudal auditory cortex (Romanski et al., 1999a,b). Furthermore, direction selective responses to visual motion have been demonstrated in this region (Zaksas and Pasternak, 2006), and like LIP, PFC neurons show activity that is consistent with accumulating sensory evidence to form perceptual decisions (Kim and Shadlen, 1999). Cells in the ventrolateral subdivision of the PFC, such as area 12, have been shown to integrate audiovisual cues, but like STP, are generally associated with higher level sensory processing, responding to individual faces and calls (Romanski, 2007, 2012). However, human imaging studies of audiovisual motion have generally not reported comparable activation in the PFC (Lewis et al., 2000; Baumann and Greenlee, 2007; von Saldern and Noppeney, 2013), although audiovisual biological motion can modulate activity in premotor areas (areas 6R and 44) when there is a mismatch between the auditory and visual cues (Wuerger et al., 2012).

A number of imaging studies have also found that audiovisual stimulation produces distinct activation (compared to visual only stimulation) in hMT+ (Alink et al., 2008; Lewis and Noppeney, 2010; Strnad et al., 2013; von Saldern and Noppeney, 2013), suggesting that auditory stimuli can modulate visually evoked responses (although this is not always the case, e.g., Wuerger et al., 2012). These regions receive sparse inputs from auditory cortex (Palmer and Rosa, 2006), and show evoked potentials in response to auditory stimuli (Chaplin et al., 2018; Kafaligonul et al., 2018). Additionally, auditory motion has been shown to affect various aspects of visual perception, such as improving visual motion detection (Kim et al., 2012), improve learning in visual motion tasks (Seitz et al., 2006), and induce visual illusions (Sekuler et al., 1997; Meyer and Wuerger, 2001; Kitagawa and Ichihara, 2002; Beer and Röder, 2004; Soto-Faraco et al., 2005; Freeman and Driver, 2008; Alink et al., 2012a; Kafaligonul and Stoner, 2012; Kafaligonul and Oluk, 2015). Altogether, these studies suggest that auditory stimuli, especially when moving, could modulate responses to visual stimuli in MT/MST.

**Figure 2:**
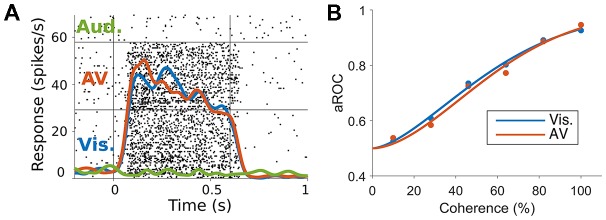
**(A)** Responses of a marmoset MT neuron to visual, auditory and audiovisual stimuli. The raster plots (black dots) and spike rate functions (colored lines) show a clear response to visual but not auditory stimuli (blue vs. green lines). The combination of auditory and visual stimuli (red line) was not significantly different to the visual only response (blue vs. red lines). **(B)** Neurometric performance (measured as the area under the receiver operating characteristic (ROC) curve, Britten et al., 1992, which corresponds to the performance of an ideal observer discriminating the direction of motion using the spiking activity of the neuron) of a marmoset MT neuron when discriminating leftwards and rightwards motion under visual (blue) and audiovisual (red) conditions at different levels of motion coherence (strength of motion signal). The addition of the auditory stimulus did not shift the neurometric curve to the left as would be expected if the neuron was integrating the auditory motion cue (adapted from Chaplin et al., 2018).

To specifically test this hypothesis, we have investigated if auditory motion cues are integrated with visual motion cues in MT/MST, by recording spiking activity and characterizing the ability of neurons to encode the direction of motion, using ideal observer analysis (Chaplin et al., 2018). We presented random dot patterns that moved either leftwards or rightwards, and manipulated the strength of the visual motion signal by reducing the coherence of the dots (i.e., making some proportion of the dots move in random directions). Reducing motion coherence reduces the both the psychophysical performance of observers (i.e., makes it more difficult to discriminate the directions of motion) and the neurometric performance of single neurons (i.e., reduces the neuronal information; Newsome et al., 1989). We hypothesized that the addition of an auditory stimulus that moved in the same direction as the visual stimulus would increase the information carried by single neurons and therefore increase neurometric performance, just as it can increase psychophysical performance in humans (Meyer and Wuerger, 2001; Kim et al., 2012). In particular, we predicted that auditory cues would be most likely be integrated at low motion coherence levels, in line with Bayesian models of multisensory integration (Ernst and Banks, 2002; Ma et al., 2006; Gu et al., 2008). However, we found no evidence of spike rate modulations (Figure 2A) or improvements in neurometric performance (Figure 2B) due to the auditory stimulus, in MT or MST. It may be the case that the audiovisual responses observed in hMT+ are the result of task related signals (Alink et al., 2012b; Bizley et al., 2016; Kayser et al., 2017), such as the binding of the two modalities to form a unified percept (Nahorna et al., 2012, 2015; Bizley and Cohen, 2013), attentional effects (Beer and Röder, 2004, 2005; Lakatos et al., 2008), or choice-related signals from the decision making process (Cumming and Nienborg, 2016).

Only one other study has investigated the effects of auditory stimuli on the responses of MT neurons (Kafaligonul et al., 2018). This study aimed to test if the activity of MT neurons mediated the temporal ventriloquist illusion, in which stationary auditory clicks induce influence the perception of visual speed. The authors hypothesized that the auditory clicks would alter the speed tuning and response duration of MT neurons in response to apparent visual motion. However, the auditory stimuli did not alter speed tuning or response duration in a way that would support the perception of the illusion, even though there was a possible modulation of the temporal spiking response. Therefore, electrophysiological studies in monkeys so far suggest that auditory stimuli do not influence visual motion perception through changes in activity to MT/MST neurons. However, since the projections from auditory to visual cortex are known to arrive at the peripheral representation of the visual field (Palmer and Rosa, 2006; Majka et al., 2018), it possible that their role of auditory inputs to facilitate the detection and localization of visual features, especially for orienting (Perrott et al., 1993; Wang et al., 2008).

## Conclusion

In conclusion, the processing of auditory and visual motion in the primate cerebral cortex utilizes different brain areas and physiological mechanisms. While good progress has been made in identifying the cortical regions involved in processing auditory and audiovisual motion, the mechanisms of audiovisual integration remain unclear. The current evidence from single neuron studies suggests that the integration of auditory and visual motion cues is not mediated by the early visual areas MT and MST, and therefore such integration likely occurs in higher level cortical areas. Another possibility is that the integration of audiovisual motion signals is not mediated by a single brain region, but instead by synchronized network activity (Lewis and Noppeney, 2010).

## Author Contributions

TC wrote the first draft of the manuscript. MR and LL wrote sections of the manuscript. All authors contributed to manuscript revision, read and approved the submitted version.

## Conflict of Interest Statement

The authors declare that the research was conducted in the absence of any commercial or financial relationships that could be construed as a potential conflict of interest.
